# Integrated Insight into the Molecular Mechanisms of Spontaneous Abortion during Early Pregnancy in Pigs

**DOI:** 10.3390/ijms22126644

**Published:** 2021-06-21

**Authors:** Xupeng Zang, Ting Gu, Wenjing Wang, Chen Zhou, Yue Ding, Shengchen Gu, Zhiqian Xu, Yanshe Xie, Zicong Li, Gengyuan Cai, Bin Hu, Linjun Hong, Zhenfang Wu

**Affiliations:** 1 National Engineering Research Center for Breeding Swine Industry, College of Animal Science, South China Agricultural University, Guangzhou 510642, China; xupeng_zang@stu.scau.edu.cn (X.Z.); tinggu@scau.edu.cn (T.G.); wenjing_wang@stu.scau.edu.cn (W.W.); czhou@stu.scau.edu.cn (C.Z.); dingyue@stu.scau.edu.cn (Y.D.); 15153918918@stu.scau.edu.cn (S.G.); xuzhiqian@stu.scau.edu.cn (Z.X.); xys@stu.scau.edu.cn (Y.X.); lizicong@scau.edu.cn (Z.L.); cgy0415@scau.edu.cn (G.C.); 2 Guangdong Provincial Key Laboratory of Agro-Animal Genomics and Molecular Breeding, College of Animal Science, South China Agricultural University, Guangzhou 510642, China; 3 Lingnan Guangdong Laboratory of Modern Agriculture, Guangzhou 510642, China; 4 State Key Laboratory for Conservation and Utilization of Subtropical Agro-Bioresources, Guangzhou 510642, China; 5 Institute of Animal Science, Guangdong Academy of Agricultural Sciences, Guangzhou 510642, China

**Keywords:** ceRNA, HOXA-AS2, immune response, pig, spontaneous abortion

## Abstract

Due to the high rate of spontaneous abortion (SAB) in porcine pregnancy, there is a major interest and concern on commercial pig farming worldwide. Whereas the perturbed immune response at the maternal–fetal interface is an important mechanism associated with the spontaneous embryo loss in the early stages of implantation in porcine, data on the specific regulatory mechanism of the SAB at the end stage of the implantation remains scant. Therefore, we used high-throughput sequencing and bioinformatics tools to analyze the healthy and arresting endometrium on day 28 of pregnancy. We identified 639 differentially expressed lncRNAs (DELs) and 2357 differentially expressed genes (DEGs) at the end stage of implantation, and qRT-PCR was used to verify the sequencing data. Gene set variation analysis (GSVA), gene set enrichment analysis (GSEA), and immunohistochemistry analysis demonstrated weaker immune response activities in the arresting endometrium compared to the healthy one. Using the lasso regression analysis, we screened the DELs and constructed an immunological competitive endogenous RNA (ceRNA) network related to SAB, including 4 lncRNAs, 11 miRNAs, and 13 genes. In addition, Blast analysis showed the applicability of the constructed ceRNA network in different species, and subsequently determined HOXA-AS2 in pigs. Our study, for the first time, demonstrated that the SAB events at the end stages of implantation is associated with the regulation of immunobiological processes, and a specific molecular regulatory network was obtained. These novel findings may provide new insight into the possibility of increasing the litter size of sows, making pig breeding better and thus improving the efficiency of animal husbandry production.

## 1. Introduction

Spontaneous abortion (SAB) of embryos in porcine pregnancy remains a global and primary concern in commercial pig farming. Previous studies have shown that around 20–30% of porcine embryos are lost between days 12 and 30 of pregnancy during the peri-implantation stage [1]. Due to the presence of a unique non-invasive epitheliochorial placenta in the porcine animals [2], post-attachment development and growth of the embryos require precise interaction with the endometrium. This interaction demands proper and abundant coordination of transcription factors, cytokines, chemokines, and hormones [3,4]. Previous studies have shown that specific chemokines and their receptors coordinate the enrichment of immune cells and their localization at the maternal–fetal interface around gestation day 15 [5,6], which is essential in supporting the critical processes of maternal–fetal adaptations. Besides, deficits in endometrial vascular remodeling that coincide with immune cell recruitment appear to be associated with retardation of the development retardation and spontaneous loss of the embryo [7,8,9]. Whereas the perturbed immune response at the maternal–fetal interface is an important mechanism associated with the spontaneous embryo loss in the early stages of implantation in porcine [8,10], data on the specific regulatory mechanism of the SAB at the end stage of the implantation remains scant.

**Figure 1:**
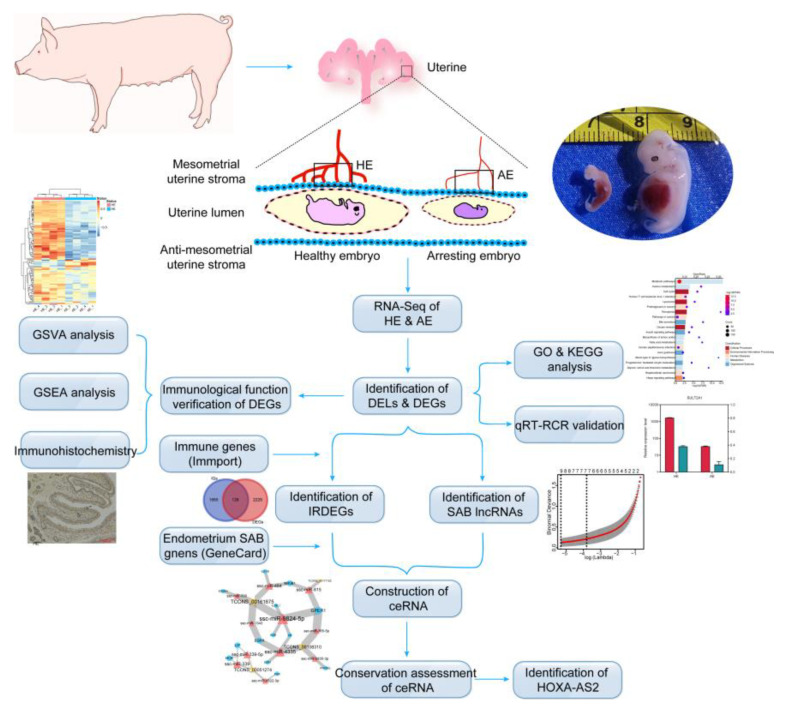
Flowchart of the study. The photograph of the embryos corresponds with the pattern diagrams, which demonstrate the healthy or arresting embryos at day 28 of pregnancy. Details of the methods and results are described later.

Long non-coding RNAs (lncRNAs) are a large class of RNAs with a nucleotide length of >200, low conservation and expression level, and tissue specificity [11]. Multiple lncRNA-specific regulatory mechanisms have been reported to influence transcription and post-transcriptional events, such as direct transcription regulation or miRNA-mediated indirect regulation of competitive endogenous RNA (ceRNA) network [12,13]. Recent studies have demonstrated that lncRNAs could regulate the expression of transcription factors, thus regulating biological processes [14]. Besides, lncRNAs have been shown to play key roles in embryo development during early pregnancy stages [15], while accumulating evidence indicates that lncRNAs, such as metastasis-associated lung adenocarcinoma transcript 1 (MALAT1), mediate angiogenesis, and could enhance neovascularization after myocardial infarction [16]. However, whether the lncRNAs could modulate the adaptations of the maternal–fetal interface during pregnancy and the exact molecular mechanism during the SAB of the embryos is yet to be determined. Here, gilts on day 28 of pregnancy (end of implantation), which is accompanied by the high level of pregnancy loss [17,18], were selected for this study, we showed data from an integrated analysis of the health and arresting conceptus attachment site (CAS), with insights into the molecular mechanism of SAB during early pregnancy in pigs (Figure 1). We provided novel information that may increase the litter size of sows and make pigs breeding better, thereby improving the efficiency of animal husbandry production.

## 2. Results

### 2.1. Genome-Wide Identification and Characterization of the lncRNAs and mRNAs

**Figure 2:**
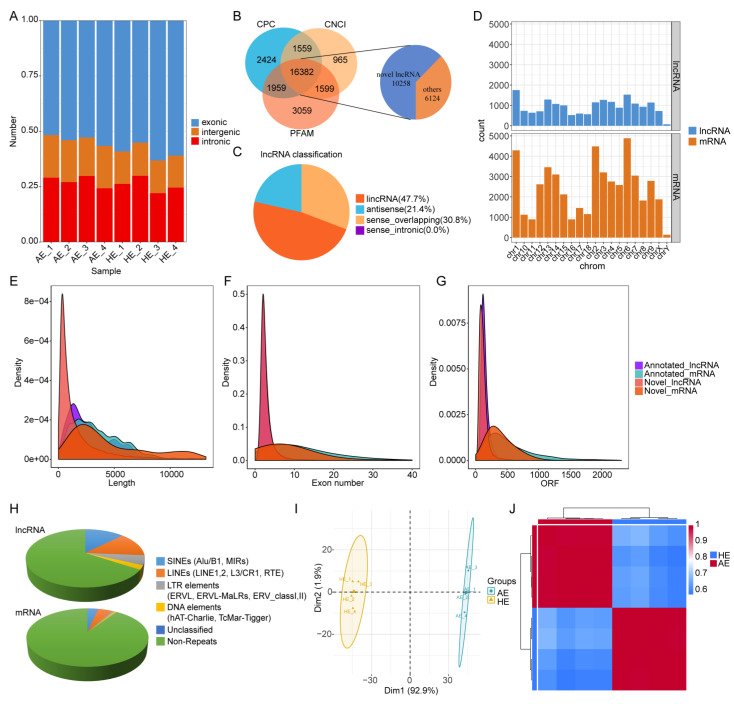
Identification and characterization of lncRNAs and mRNAs. (**A**) Distribution of the reads in different regions of the genome. (**B**) Screening of candidate novel lncRNAs. Three tools (CPC, CNCI, and PFAM) were used to analyze the coding potential of the lncRNAs, and the iterated lncRNAs were designated as candidate novel lncRNAs and used together with annotated lncRNAs for subsequent analysis. (**C**) Classification of the novel lncRNAs. (**D**) Distribution of lncRNAs and mRNAs in the genome. (**E**–**G**) Transcript length, exon number, and ORF length distribution of the lncRNAs and mRNAs. (**H**) Pie chart showing the percentage distribution of repeat sequences in the lncRNA and mRNA populations. (**I**,**J**) PCA and hierarchical clustering heatmap were used to check the differences between paired endometrial samples.

A total of 701,720,602 raw reads were generated from the 4 paired endometrium porcine samples using Illumina Hiseq 2500 platform. Out of the total raw reads, 695,694,564 were clean (Supplementary Table S2), accounting for 99% of raw reads. Mapping of these reads to the genome showed that most of them were in the exon region (Figure 2A). Further analysis of the coding potential using CNCI, CPC, and Pfam software identified 10,258 novel lncRNAs, representing 52.3% of all the identified lncRNAs (Figure 2B). Of the novel lncRNAs, 47.7%, 30.8%, or 21.4% were long intergenic non-coding RNAs (lincRNAs), sensed overlapping lncRNAs or antisense lncRNAs, respectively (Figure 2C). Besides, analysis of genomic location distribution showed that the lncRNAs were mainly distributed on chromosomes 1 and 6, while the mRNAs were mainly on chromosomes 1, 2 and 6. Whereas the location of the mRNAs was approximately the same as the distribution of the lncRNAs, their abundance was more than that of the lncRNAs (Figure 2D).

To further understand the differences between the lncRNAs and mRNAs in the HE or AE, we compared and analyzed data on gene structure and expression patterns. Notably, both the length of the lncRNAs and the number of exon and open reading frames (ORF) of the lncRNAs (Figure 2E,F) were shorter than that of the mRNAs (Figure 2G). The average length of the lncRNA and mRNA was 2430.95 and 3523.44 nt, respectively. Consistent with previous reports [19], our data showed that the lncRNA was richer with repeat sequences compared with the mRNA, including 12.70% short interspersed nuclear elements (SINES), 13.60% long interspersed nuclear elements (LINES), 5.40% long terminal repeats (LTR), 2.38% DNA elements, and 0.02% unclassified. There were 65.90% non-repetitive sequences (Figure 2H), which indicated that the lncRNAs might be playing specific biological functions in evolution. In addition, Figure 2I,J shows that the distance within the same category group is closer. The data indicated a correlation within the paired sample group (Supplementary Table S3).

### 2.2. Differentially Expressed LncRNAs (DELs) and Their Functions

**Figure 3:**
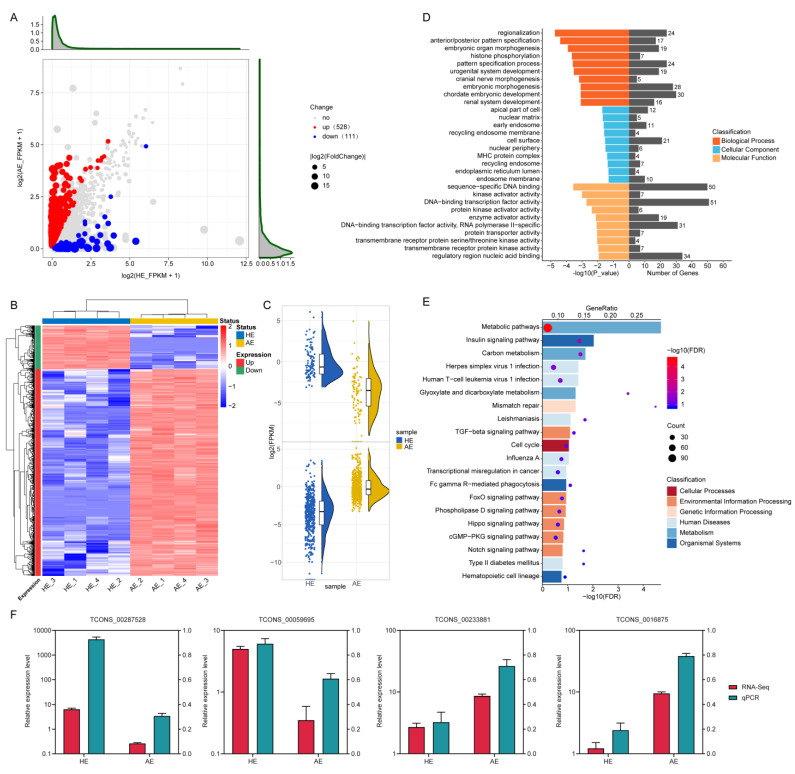
Screening and enrichment analysis of the differentially expressed lncRNAs (DELs) in AE compared with HE. (**A**) DELs expression profile by scatter plot. Each point represents one lncRNA. The red points represent upregulated lncRNAs while the blue points represent downregulated lncRNAs. (**B**) Hierarchical clustering heatmap of the DELs. The color scale is from −2.0 (blue, lower lncRNA expression level) to 2.0 (red, higher lncRNA expression level). Each row represents one lncRNA, and each column represents one sample. The red band on the left side of the heatmap represents clustering of the upregulated lncRNAs, and the green represents the downregulated lncRNAs. (**C**) Raincloud plot of the upregulated and downregulated DELs. (**D**) GO enrichment analysis of the DELs nearest target genes. (**E**) KEGG pathway analysis of the DELs nearest target genes. The different color represents the categories to which the KEGG pathway belongs. (**F**) Validation of the expression of genes using qRT-PCR. The relative expression level was normalized by log10.

Using an FDR < 0.05 and |FoldChange| > 2, we identified 639 DELs in both the HE and AE. There were 528 upregulated and 111 downregulated DELs (Figure 3A, Supplementary Table S4). Hierarchical clustering analysis and raincloud plot showed lncRNAs expression patterns among the samples (Figure 3B,C). To identify the function of the DELs, we performed GO enrichment and KEGG pathway analyses on the lncRNA nearest target genes. Data from the KEGG pathway analysis showed significant enrichment of many immune-related pathways, such as ‘Herpes simplex virus 1 infection’, ‘Human T-cell leukemia virus 1 infection’, and ‘Leishmaniasis’. Thus, the DELs might be mediating immune functions (Figure 3E, Supplementary Table S5). On the other hand, the GO enrichment analysis showed that the DEL nearest target genes were enriched in some biological processes, such as ‘regionalization’, ‘anterior/posterior pattern specification’, and ‘histone phosphorylation’ (Figure 3D, Supplementary Table S5). We then randomly selected 4 DELs for validation using qRT-PCR (Figure 3F, Supplementary Table S6), and the data consistent with the results obtained from the bioinformatics analyses.

### 2.3. Analysis of Differentially Expressed Genes (DEGs) and Their Functions

**Figure 4:**
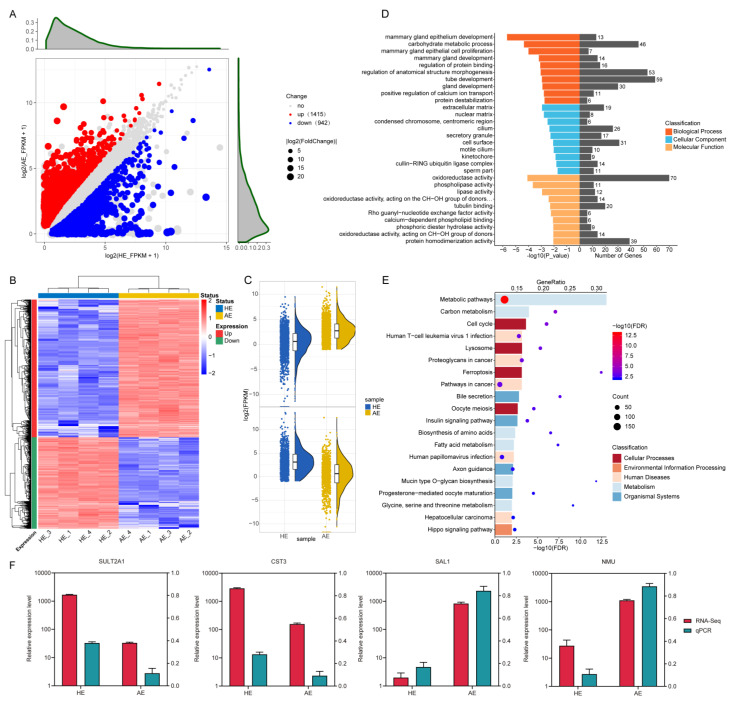
Screening and enrichment analysis of the differentially expressed genes (DEGs) in AE compared with HE. (**A**) DEGs expression level analysis by scatter plot. Each point represents one gene. The red points represent upregulated genes while the blue points represent downregulated genes. (**B**) Hierarchical clustering heatmap of the DEGs. The color scale is from −2.0 (blue, lower gene expression level) to 2.0 (red, higher gene expression level). Each row represents one gene, and each column represents one sample. The red band on the left side of the heatmap represents the clustered upregulated genes, while the green represents downregulated gene clusters. (**C**) Raincloud plot of upregulated and downregulated DEGs. (**D**) GO enrichment analysis of the DEGs. (**E**) KEGG pathway analysis of the DEGs. The different color represents the categories to which the KEGG pathway belongs. (**F**) Validation of the expression of genes using qRT-PCR. The relative expression level was normalized by log10.

Analysis of the differential expression identified 2357 DEGs, including 1415 upregulated and 942 downregulated genes (Figure 4A, Supplementary Table S4). Hierarchical clustering analysis and raincloud plot categorized the genes with significant changes into two clusters (Figure 4B,C). Similarly, we performed GO enrichment and KEGG pathways analysis on the DEGs. As demonstrated by the DELs, the significantly enriched KEGG pathways involved immune-related pathways, such as ‘Human T-cell leukemia virus 1 infection’ and ‘Lysosome’, thus signifying mediation of the immune functions (Figure 4E, Supplementary Table S5). Besides, the GO functional analysis showed that the DEGs were associated with development processes (Figure 4D, Supplementary Table S5). Similarly, the RNA-sea data on the 4 DEGs was randomly selected and validated by qRT-PCR (Figure 4F, Supplementary Table S6).

### 2.4. Weakened Immunobiological Activities in AE

**Figure 5:**
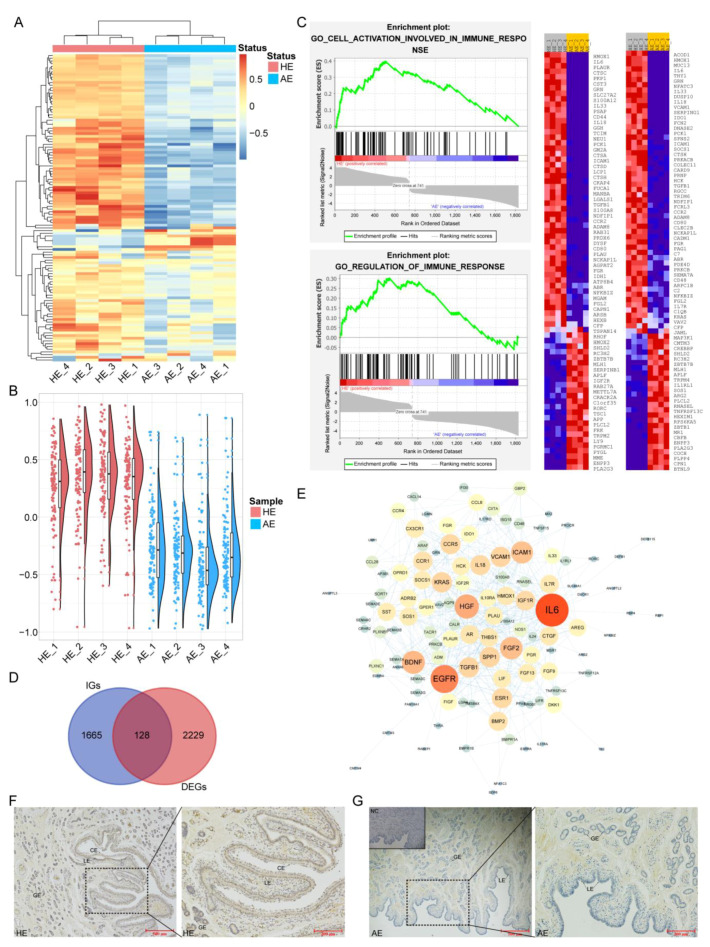
Weakened immunobiological activities in AE. (**A**) Gene set variation analysis (GSVA) demonstrated enrichment of the immunobiological processes in HE. Each row represents one biological process, and each column represents one sample. (**B**) Raincloud plot of the GSVA enrichment results. (**C**) Gene set enrichment analysis (GSEA) indicated a positive enrichment of two immunobiological processes (‘cell activation involved in immune response’ and ‘regulation of immune response’) in HE. The heatmap on the right shows the gene expression level in the two biological processes of enrichment. (**D**) Venn diagram of immune genes (IGs) and DEGs, the intersection indicates the differentially expressed genes related to immunity. (**E**) PPI network of IRDEGs visualized using Cytoscape. The size of the circle represents the degree of interaction between the genes. Immunohistochemical localization of CD44 in HE (**F**) and AE (**G**). CD44 expression was evident in HE, while almost not expressed in AE. CE: chorionic epithelium; GE: glandular epithelium; LE: luminal epithelium.

To further determine whether the DEGs between the AE and HE were related to the immunological functions, 139 immunobiological process gene sets were downloaded from MSigDB, and then gene set variation analysis (GSVA) was performed based on 139 GO terms (Supplementary Table S7). The two different states of the endometrium had significant differences in the GSVA, and the immunobiological processes were enriched in the HE (Figure 5A,B). Furthermore, the gene set enrichment analysis (GSEA) of the immunobiological processes showed a significant positive enrichment of ‘cell activation involved in immune response’ and ‘regulation of immune response’ in the HE group (Figure 5C, Supplementary Figure S1). The results of immunohistochemistry proved that CD44 was significantly expressed in HE (Figure 5F,G). Overall, our data demonstrated that these DEGs are involved in immunobiological processes, and weakened immunobiological activities in AE.

In addition, we downloaded a total of 1793 immune genes from Immport (Supplementary Table S8), an immune database, and then obtained 128 immune-related (IR) DEGs (Figure 5D). Analysis of the protein–protein interaction (PPI) networks of the IRDEGs using the STRING database showed that IL6, EGFR, HGF, BDNF, and FGF2 were key nodes of the IRDEGs (Figure 5E).

### 2.5. Construction of Immunological ceRNA Network Related to the SAB

**Figure 6:**
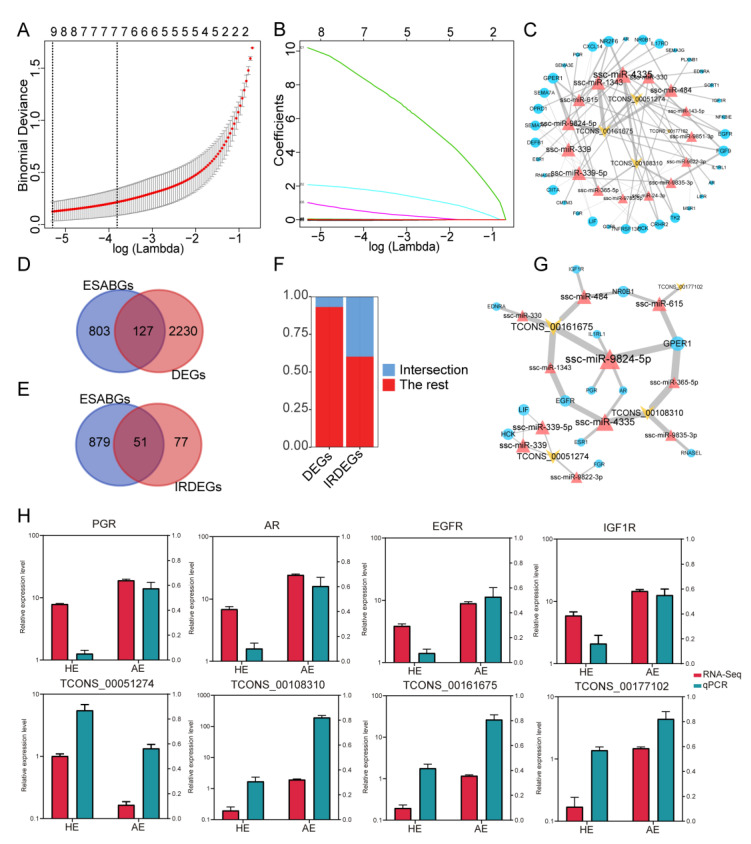
The immune-related endometrial spontaneous abortion (SAB) competitive endogenous RNA (ceRNA) network. (**A**) Results of the lasso regression analysis for 639 DELs. Ten-fold cross-validation was used to calculate the best lambda value that results in the minimum mean cross-validation error. The red dot represents partial likelihood deviation, while the vertical solid line represents its corresponding 95% confidence interval. (**B**) The coefficient values at varying levels of penalty. Each curve represents an lncRNA. (**C**) The immune-related ceRNA network. The yellow quadrilateral represents lncRNA, the red triangle represents miRNA, while the blue circle represents genes. The degree of connection of the nodes is indicated by the size of the shape and the thickness of the edge. (**D**) Venn diagram of endometrial SAB genes (ESABGs) and DEGs. (**E**) Venn diagram of ESABGs and immune-related differentially expressed genes (IRDEGs). (**F**) The bar chart shows that EASG has a larger proportion in the IRDEG. (**G**) The immune-related endometrial SAB ceRNA network. The yellow quadrilateral represents lncRNA, the red triangle represents miRNA, while the blue circle represents genes. The degree of connection of the nodes is indicated by the size of the shape and the thickness of the edge. (**H**) Relative expression of the DELs and several DEGs in the ceRNA network.

To identify the lncRNA implicated in SAB, we performed a lasso regression analysis of the DELs and identified the following 8 lncRNAs: ENSSSCT00000004780, TCONS_00042638, TCONS_00051274, TCONS_00071236, TCONS_00108310, TCONS_00161675, TCONS_00177102, and TCONS_00215223 (Figure 6A,B). We then predicted the target miRNAs for these 8 lncRNAs, and estimated the target miRNAs for the 128 IRDEGs. The overlapping miRNAs were selected for the construction of the ceRNA network. Given the potential correlation between the lncRNAs and the genes in the ceRNA network, we conducted a correlation analysis and selected lncRNA and gene pairs with a correlation coefficient of >0.8 (Supplementary Table S9). As a result, we obtained an immunological ceRNA network comprising 52 unique RNAs (4 lncRNAs, 15 miRNAs, and 33 genes), as shown in Figure 6C.

In addition, in order to evaluate whether the constructed immunological ceRNA network was related to SAB, we downloaded 930 endometrial spontaneous abortion genes (ESABGs) from the GeneCard database (Supplementary Table S8). The data showed that the proportion of these genes in the IRDEGs was significantly higher than in the DEGs, indicating that the SAB process was probably triggered by the overlapping immune genes (Figure 6D,F). We further constructed the SAB ceRNA network from the immunological ceRNA network (4 lncRNAs, 11 miRNAs, and 13 genes) (Figure 6G). The related DELs and several DEGs that were identified in the ceRNA network were verified by the qRT-PCR (Figure 6H), demonstrating the robustness of the constructed ceRNA network.

### 2.6. Evaluation of the Applicability of SAB ceRNA Network among Animals Species

**Figure 7:**
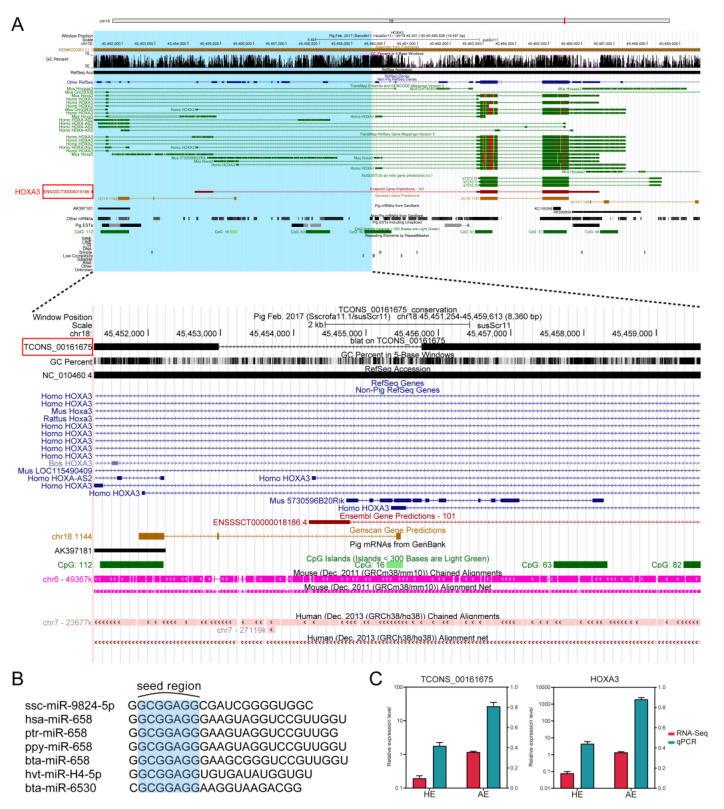
Assessment of lncRNA and miRNA conservation. (**A**) Schematic diagram of the 45,451,130–45,469,626 bp genome annotation of pig chromosome 18, depicted with the gene loci of HOXA3 and the identified lncRNA TCONS_00167675, which is highlighted by the red boxes. (**B**) Alignment of ssc-miR-9824-5p with miRNAs of different species in the seed region. (**C**) Relative expression levels of TCONS_00161675 and HOXA3.

Due to the low conservation of the lncRNAs, using lncRNAs identified in different species are generally not extrapolated for other species. To assess whether our constructed ceRNA mechanism for SAB of the embryos could be applied to different species, we analyzed the sequence conservation properties of the lncRNA TCONS_00161675, with the most connectivity in the ceRNA network using NONCODE BLAST. Surprisingly, we found a 716 bp region matching with NONHSAT211839.1 (human lncRNA), and 650 bp with NONMMUT056947.2 (mouse lncRNA) (Supplementary Table S10). This demonstrated that the TCONS_00161675 has an extremely high species conservation properties and may play important roles in evolution. To further understand of the localization of the TCONS_00161675 conserved region on the genome, the UCSC genome browser was used to map the location with surrounding genes (Figure 7A). Interestingly, we found that the identified lncRNA is an antisense lncRNA of the transcription factor, HOXA3. Compared with the known Homo HOXA-AS2, we associated it with HOXA-AS2 in pigs. In addition, the expression of HOXA-AS2 and HOXA3 were found to be simultaneously upregulated compared with those in the HE (Figure 7C), and thus might be playing a common function.

Previous studies have shown that miRNAs function mainly through the seed sequences at nucleotide positions 2 through 7. Besides the seed sequences indicating that the miRNAs target genes could be the same (Figure 7B) [20], overall, the SAB ceRNA network with a highly conservative element composition could be applied in different animal species.

## 3. Discussion

Porcine animals are a unique SAB model, where up to 45% of embryos are spontaneously lost as embryos develop, which allows detailed study of the specific molecular regulation mechanisms [21]. The high-throughput RNA-seq of the HE or AE presents an opportunity to improve our understanding of the molecular regulation of SAB. In this study, we identified some lncRNAs and mRNAs involved in SAB and demonstrated that SAB is related to the immunobiological processes, which is significantly weakened in the arresting state. Subsequently, we constructed an immunological ceRNA network related to SAB, and showed that it might be applicable to different animal species.

The implantation in pigs begins on days 15–16 of pregnancy and ends on days 27–28 of pregnancy. Previous studies have shown that around 20–30% of porcine embryos are lost between days 12 and 30 of pregnancy [1]. In addition to the embryo loss caused by the rapid conceptus elongation during the peri-implantation stage [22], another peak of embryo loss is from the postattachment to the end of implantation, that is, days 15–28 of pregnancy, which is accompanied by the maternal angiogenesis and endometrial lymphocytes recruitment [6,7,18,23]. Tayade et al. analyzed the endometrium, endometrial lymphocytes, and trophoblasts from days 15–23 of pregnancy to prove that blocked maternal angiogenesis and the abnormal expression of proinflammatory factors leads to spontaneous fetal loss [24]. However, data on the specific regulatory mechanism of the spontaneous abortion at the end stage of the implantation remain scant. Therefore, we comprehensively considered these factors and finally selected day 28 of pregnancy at the end of implantation for this study.

Previous studies associated the loss of embryo with recruitment of lymphocytes and angiogenesis in the porcine maternal–fetal interface during the early stage of implantation. Whereas there was increased transcription of angiogenic genes in healthy implantation sites, the arresting fetal sites showed rapid elevation in the expression of proinflammatory cytokines, Fas and Fas ligands [24]. Although our results showed that SAB of embryos on day 28 of pregnancy was related to immunological processes, the immunobiological activities were higher in HE compared to the AE, while the expression of angiogenetic genes was downregulated. The higher immune response activities in HE might be due to the differences in the epitheliochorial placenta between the porcine and humans or rodents. The porcine placenta forms through the attachment of chorion to the maternal epithelium and six tissue layers separate maternal and fetal blood supplies [25]. To avoid maternal rejection of the semiallogeneic embryo, it is necessary to regulate the immune response activities for normal embryo development [6,26]. The high expression of angiogenetic genes in AE could be due to the completion of embryo implantation on day 28 of pregnancy [17], and the development of vascular system of the healthy endometrium to a level that could maintain further embryo development.

In addition, growing evidence indicated that many lncRNAs play a vital role in the regulation of cellular growth and disease development [27,28]. As an important molecular regulatory mechanism for lncRNAs in exerting biological functions, ceRNA has attracted immense research interest in the recent years [29]. Recent studies have shown that aberrant expression of lncRNAs can lead to SAB [30]. Similarly, in this study, we have identified 8 SAB-related lncRNAs. Further, to clarify the biological implications of the expression of these lncRNAs in SAB, we constructed an immunological ceRNA network, which included 4 novel lncRNAs (TCONS_00051274, TCONS_00108310, TCONS_00161675, and TCONS_00177102), 11 miRNAs, and 13 genes. However, the expression of the lncRNAs was generally tissue and time-specific, and showed a low level of expression and sequence conservation [31,32]. Thus, the previously constructed ceRNA was not applicable to other species. To our surprise, the lncRNAs in the SAB ceRNA network we constructed were highly conserved among different animal species, and the miRNAs were evolutionarily conserved [33]. Our constructed ceRNA network was, therefore, more likely to be applicable to the other species. This provides a valuable animal model for studying SAB in other species, such as humans.

Interestingly, our analysis found that the highly conserved lncRNA TCONS_00161675 overlaps with the transcription factor HOXA3 in the genomic location. Compared with the known Homo HOXA-AS2, we associated the conserved gene with HOXA-AS2 in pigs. Previous studies have shown that HOXA3 plays an important role in embryonic development, including control of distinct genetic programs for differentiation and morphogenesis in different cell types [34,35]. In addition, HOXA-AS2 can regulate the expression of HOXA3 to perform specific biological functions in human [36,37]. Therefore, HOXA-AS2 may also regulate the expression of HOXA3 in pigs in the execution of critical biological processes.

## 4. Materials and Methods

### 4.1. Ethics Statement and Sample Collection

This study was approved by the Ethics Committees of the Laboratory Animal Center of South China Agricultural University (permit number: SYXK-2019-0136).

Tibetan gilts with an average litter size of 7–8, which have been artificially fed and bred for multiple generations on farms in Guangdong, China, were slaughtered at a local slaughterhouse on day 28 of pregnancy and the uteri were quickly removed and transported to the laboratory in an icebox. The uteri samples were then opened longitudinally along the anti-mesometrial side and the healthy and arresting embryos were analyzed. The embryos were obtained from 4 different pigs and the analysis was based on embryo length, weight, and vascularity of the placental membranes (Figure 1). Subsequently, the embryos were exposed for visual classification of the healthy and arresting CAS. The ratio between healthy and arresting embryos was around 3:1–4:1. After removing the embryos, the healthy endometrium (HE) and arresting endometrium (AE) samples were collected, immediately immersed in liquid nitrogen, and then transferred to a −80 °C freezer for subsequent RNA extraction.

### 4.2. RNA Isolation, Library Construction, and Sequencing

Total RNA of endometrium tissues was extracted using TRIzol reagent (Invitrogen, Carlsbad, CA, USA) following the manufacturer’s instructions. The RNA purity and concentration were assessed using NanoPhotometer^®^ spectrophotometer (IMPLEN, Westlake Village, CA, USA). Furthermore, RNA integrity and quantity were measured using the RNA Nano 6000 Assay Kit of the Bioanalyzer 2100 system (Agilent Technologies, Santa Clara, CA, USA), and the RIN (RNA integrity number) values were above 7.60.

The library was prepared by an rRNA depletion method [38], and constructed and sequenced by Novogene Co. Ltd. (Beijing, China). Specifically, the ribosomal RNA was depleted from total RNA using the Epicentre Ribozero™ rRNA Removal Kit (Epicenter, Madison, WI, USA) following the manufacturer’s protocol. Subsequently, the RNA was fragmented into 250–300 bp, and the fragmented RNA and dNTPs (dATP, dTTP, dCTP, and dGTP) were used for reverse transcription of the first-strand cDNA. RNA was degraded using RNase H, and second-strand cDNA was synthesized using DNA polymerase I and dNTPs. Then, through the exonuclease/polymerase activities, the remaining overhangs of double-stranded cDNA were converted to blunt ends. After the 3′ ends of the DNA fragments were adenylated, sequencing adaptors were ligated to the cDNA. The library was purified by using the AMPure XP system to select cDNA fragments preferably 350–400 bp. Uridine digestion was performed using Uracil-N-Glycosylase, which was followed by the cDNA amplification using PCR.

After the library was constructed, the concentration of the library was adjusted to 1 ng/ul. Agilent 2100 Bioanalyzer was deployed to detect the insert size of the acquired library. Last, qPCR was used again to check the exact concentration of the cDNA library. The library preparation was completed, and the libraries were sequenced on Illumina PE150.

### 4.3. Quality Control and Transcriptome Assembly

Raw reads were trimmed by removing adapter sequences, reads with more than 10% ploy-N, reads with ploy-A/T/G/C, and reads with more than 50% nucleotides with Qphred ≤ 20. Besides, Q20, Q30, and GC contents of the clean reads were calculated. All downstream analyses were based on the high-quality clean reads. Clean reads for each sample were mapped to the reference genome (Sscrofa11.1, http://asia.ensembl.org/Sus_scrofa/Info/Index, accessed on 3 October 2020) with the software HISAT2 (v.2.0.5) and the reads alignment results were transferred to StringTie (v.1.3.3) for transcript assembly [39,40].

### 4.4. LncRNA Identification and Characterization

All transcripts were merged using Cuffmerge (v.2.2.1) software, and then, based on the characteristics of lncRNAs, lncRNAs were identified from the assembled transcripts. First of all, the transcripts with FPKM < 0.1, length < 200 bp, and exon numbers < 2 were removed. Cuffcompare (v.2.2.1) was used to compare transcripts with the reference to remove the annotated transcripts. Whereafter, we analyzed the coding potential of the transcripts by three software (CNCI (v.2) [41], Pfam (v.1.3) [42], and CPC2 (v.3.2.0) [43]) and eventually identified novel lncRNAs. The repeat sequences were extracted using the default parameters in Repeatmasker (v.2.10.0+) [44].

### 4.5. Differential Expression Analysis and Function Enrichment Analysis

Fragments per kilobase for a million reads (FPKM) of lncRNAs and mRNAs was calculated by StringTie (v1.3.3). Then, a paired t-test was used to analyze the differential expression of lncRNAs and genes, and the *p*-value was corrected by the FDR method [45]. FDR < 0.05 and |FoldChange| > 2 was set as the threshold of significant differential expression between AE and HE. Based on the gene expression data, principal component analysis (PCA) was performed to analyze the similarity of the samples. In addition, we calculated the Pearson coefficient to identify the differences within the paired sample groups.

The genes within 100 kb upstream and downstream of lncRNAs were used as the nearest target genes of lncRNAs [46]. GO enrichment analysis of target genes of differentially expressed lncRNAs (DELs) and differentially expressed genes (DEGs) were performed using the clusterProfiler R package (v.3.10.1) [47] and KEGG pathway analyses were performed by the software KOBAS (v.3.0, http://kobas.cbi.pku.edu.cn/kobas3, accessed on 15 October 2020). Moreover, the protein–protein interaction (PPI) network was analyzed using STRING database (https://string-db.org, accessed on 23 October 2020), and Cytoscape (v.3.7.2, https://cytoscape.org, accessed on 23 October 2020) was used for further visualization.

### 4.6. Quantitativereal-Time RT-PCR

The total RNA isolated from the endometrium tissue was subjected to quantitative real-time PCR (RT-PCR). Briefly, we synthesized cDNA using a PrimeScript™ RT reagent Kit with gDNA Eraser (TaKaRa, Dalian, China). We then performed quantitative RT-PCR PowerUp™ SYBR™ Green Master Mix (ThermoFisher, Shanghai, China) on the Step One Plus Real-Time PCR System (Life Technologies, Frederick, MD, USA). We adopted the following qRT-PCR protocol: 95 °C for 10 min, 50 cycles of 95 °C for 15 s, 60 °C for 15 s, and 72 °C for 20 s. Primers were designed using NCBI Primer-BLAST and Oligo 7 (http://www.oligo.net, accessed on 11 December 2020), and all reactions were run in triplicate (Supplementary Table S1). The relative expression of lncRNAs and genes were calculated with the 2^−ΔΔCt^ method and normalized using SLC39A7 and ZNF783.

### 4.7. Immunological Function Verification of DEGs

Functional gene sets of immunobiological process were obtained from the molecular signature database (MSigDB, v.7.2, https://www.gsea-msigdb.org/gsea/msigdb, accessed on 6 January 2021). Subsequently, gene set variation analysis (GSVA) and gene set enrichment analysis (GSEA) were conducted to explore the enrichment of DEGs in the functional gene sets of immunobiological processes. The immune genes (IGs) were acquired from the Immport database (https://www.immport.org/home, accessed on 7 January 2021) and the endometrium spontaneous abortion genes (ESABGs) were downloaded from the GeneCard database (https://www.genecards.org, accessed on 7 January 2021).

### 4.8. Immunohistochemistry

To evaluate the activities of immunobiological processes in HE and AE, we selected CD44, an antibody involved in a broad range of leukocyte activities which reacts with all leukocyte classes [48], to perform immunohistochemical analysis as in prior reports [49]. Briefly, 4 μm-thick uteri sections were deparaffinized and blocked with 5% BSA and then incubated with anti-CD44 mouse monoclonal antibodies (GB14037, Servicebio, Wuhan, China) at 4 °C overnight. Purified nonrelevant immunoglobulin G (IgG), at the same concentration as the corresponding primary IgG, was used as the negative control. After incubating with the secondary antibody, the sections were counterstained with hematoxylin (Fisher Scientific, Shanghai, China). The images were taken by Nikon microscope 80i with a digital camera DS-Fi1 (Nikon, Tokyo, Japan).

### 4.9. Construction of Competing Endogenous RNA (ceRNA) Network

To identify the potential lncRNAs associated with SAB, we performed a lasso regression analysis on DELs. The sequence information of miRNAs was downloaded from the miRBase database (v.22.1, http://www.mirbase.org, accessed on 5 February 2021). Based on the ceRNA hypothesis, we predicted the interaction between lncRNAs and miRNAs, and miRNAs and mRNAs by three computational target prediction algorithms (miRanda (v.3.3) [50], Targetscan (v.7.0) [51], and RNAhybrid (v.2.1.2) [52]). The correlation between lncRNAs and mRNAs was analyzed by calculating the Pearson correlation coefficient, and the *p*-value was corrected by the FDR method. Then, we selected the lncRNA-mRNA relationship pair with a correlation coefficient > 0.8 and FDR < 0.05 to construct the ceRNA network.

### 4.10. Sequence Conservation Analysis of lncRNA and miRNA

The sequence of lncRNA was submitted to the NONCODE database (http://www.noncode.org, accessed on 15 February 2021) for blast comparison analysis to understand the conservation of lncRNA among different animal species, and visualized through the UCSC genome browser (http://genome.ucsc.edu, accessed on 15 February 2021). In addition, the conservation of miRNA was comprehended by analyzing the consistency of the miRNA seed region sequence.

### 4.11. Statistical Analysis

The data are presented as the mean ± the standard error of the mean (SEM). The group data was compared using the Student’s *t*-test (GraphPad Prism version 8.0, San Diego, CA, USA). A *p*-value of less than 0.05 was considered to be statistically significant.

## 5. Conclusions

In conclusion, we profiled the whole transcriptome expression of HE and AE in porcine on day 28 of pregnancy and obtained some lncRNAs and mRNAs involved in SAB. Our data demonstrated a correlation between SAB at the completion stage of implantation and the immunobiological process, but showed different molecular mechanisms from the beginning stage of the implantation. Finally, an immunological ceRNA network related to SAB was constructed and HOXA-AS2 in pigs was identified. Further studies are, however, required to verify the constructed ceRNA network and its applicability among different species, as well as the specific molecular mechanisms of action of the identified HOXA-AS2 in pigs. These novel findings will provide new targets for increasing the litter size of sows, making pig breeding better and thus improving the efficiency of animal husbandry production.

## Data Availability

The datasets presented in this study can be found in online repositories. The raw reads produced in this study were deposited in the NCBI Sequence Read Archive (SRA). The accession number is PRJNA687816.

## Supplementary Materials

The following are available online at https://www.mdpi.com/article/10.3390/ijms22126644/s1, Supplementary Figure S1: GSEA enrichment analysis of DEGs, Supplementary Table S1: Primer sequence for the lncRNAs and genes for Real-time RT-PCR, Supplementary Table S2: Summary of high-throughput sequencing in HE and AE, Supplementary Table S3: The values of Pearson’s correlation coefficient, Supplementary Table S4: FPKM of DELs and DEGs in HE and AE, Supplementary Table S5: GO enrichment and KEGG pathway analysis of nearest target genes of DELs or DEGs, Supplementary Table S6: Validation of RNA-seq results by using qRT-PCR, Supplementary Table S7: Immunobiological process gene sets, Supplementary Table S8: Immune genes from the Immport database/endometrium spontaneous abortion genes from the GeneCard database, Supplementary Table S9: Pearson correlation coefficient of lncRNAs and genes, Supplementary Table S10: Conserved sequences of the lncRNA in different species.
