# Supplementary material for: Integrated Insight into the Molecular Mechanisms of Spontaneous Abortion during Early Pregnancy in Pigs
Source: Int J Mol Sci. 2021 Jun 21;22(12):6644. doi: 10.3390/ijms22126644 (PMC8235555; doi:10.3390/ijms22126644)
Supplement: Supplementary file 1 [file ijms-22-06644-s001.zip › Supplementary Table 10.pdf]

>NONHSAT211839.1

NONCODE TRANSCRIPT ID	NONHSAT211839.1
NONCODE Gene ID	NONHSAG047182.2
Chromosome	chr7
Start Site	27114535
End Site	27122881
Strand	+
Exon Number	3
CNCI Score	-0.0217088
Length	567
Assembly	hg38
Other transcript Versions	None

```
Query: 1008 tgcaattccaaggtgtcaagggctcggcctcctcaaaggaaggtgttaaaaatataagta 1067
            |||||| |||||| ||||| |||  | ||| |||||||||||||||||||||||||||||
Sbjct: 547  tgcaatcccaaggcgtcaacggcgggtccttctcaaaggaaggtgttaaaaatataagta 606


Query: 1068 gtgtgtctaaactgtcagggagccaaagtgtcaccttagagcaaaaacaaagccaaggga 1127
            || ||| |||||||||| ||||||||||||||||||||||||||||||||||||| ||||
Sbjct: 607  gtatgtttaaactgtcaaggagccaaagtgtcaccttagagcaaaaacaaagccagggga 666


Query: 1128 aaagggagaaatcaaaaagcactctgggccagggtggcctcatgcataccaatggttttt 1187
            |||||||||||||||||||||||| |||||||||||||||||||||||||||||||||||
Sbjct: 667  aaagggagaaatcaaaaagcactccgggccagggtggcctcatgcataccaatggttttt 726


Query: 1188 gtcatttatggctgggcaagatttatgactcggcgccccaaagctgtaaacagagcacaa 1247
            ||||||||||||||||||||||||||||||||||||||||||||||||||||||||||||
Sbjct: 727  gtcatttatggctgggcaagatttatgactcggcgccccaaagctgtaaacagagcacaa 786


Query: 1248 aacagcaaacacttgctccttatggcatattgctgcggcgcaacttgaaagggggagccg 1307
            ||||||||||||||||||||||||||||||   ||| | |||||||||||||||||| |
Sbjct: 787  aacagcaaacacttgctccttatggcatat---tgcagagcaacttgaaagggggagctg 843


Query: 1308 ggcccgcgtaggaggcaagagccggccgcaaagagccgctcagcggcattttgtcttcaa 1367
            |  ||||||||||||  ||||||  |||||||| | ||| || |||||||||| |||||||
Sbjct: 844  g--ccgcgtaggaggtgagagccagccgcaaaaatccgttcggcggcattttctcttcaa 901


Query: 1368 accggccgggtcggatgtggcatttgaagaaagaaggcgtgggtcaataatcaacccgca 1427
            ||| ||| ||||||||||||||||||||||||||||||||||||||||||||||||||||
Sbjct: 902  acccgcccggtcggatgtggcatttgaagaaagaaggcgtgggtcaataatcaacccgca 961


Query: 1428 ctttctcctccaaactgctgacgcga--ctcaccgccttctcagctaagccagctgcagg 1485
            ||||||||||||||||||||||||||  ||||||||||||||||||||||||||||| ||
Sbjct: 962  ctttctcctccaaactgctgacgcgactctcaccgccttctcagctaagccagctgccgg 1021


Query: 1486 gaaggccagctctccgtgagaacct 1510
            ||||||||||||||||| |||||||
Sbjct: 1022 gaaggccagctctccgtaagaacct 1046
```

>NONMMUT056947.2

NONCODE TRANSCRIPT ID	NONMMUT056947.2
NONCODE Gene ID	NONMMUG035351.2
Chromosome	chr6
Start Site	52175234
End Site	52215076
Strand	+
Exon Number	3
CNCI Score	-0.0679936
Length	2207
Assembly	mm10
Other transcript Versions	NONMMUT056947.1(old version)

```
Query: 987  aagagagaaatggagctttcgtgcaattccaaggtgtcaagggctcggcctcctcaaagg 1046
            |||||||||| ||||||||||||||||||| ||| ||||| |      |||| |||||||
Sbjct: 1633 aagagagaaaaggagctttcgtgcaattccgaggcgtcaacgagggtccctc-tcaaagg 1691


Query: 1047 aaggtgttaaaaatataagtagtgtgtctaaactgtcagggagccaaagtgtcaccttag 1106
            ||||||||||||||||||||||| ||| ||||||||||||||||||||||||||||||||
Sbjct: 1692 aaggtgttaaaaatataagtagtatgtttaaactgtcagggagccaaagtgtcaccttag 1751


Query: 1107 agcaaaaacaaagccaaggga-aaagggagaaatcaaaaa-gcactctgggccagggtgg 1164
            |||||||||||||||| |||  |||||||||||||||||| |||||| ||||||||||||
Sbjct: 1752 agcaaaaacaaagccaggggggaaagggagaaatcaaaaaagcactccgggccagggtgg 1811


Query: 1165 cctcatgcataccaatggtttttgtcatttatggctgggcaagatttatgactcggcgcc 1224
            ||||||||||||||||||||||||||||||||||||||||||||||||||||||||| ||
Sbjct: 1812 cctcatgcataccaatggtttttgtcatttatggctgggcaagatttatgactcggctcc 1871


Query: 1225 ccaaagctgtaaacagagcacaaaacagcaaacacttgctccttatggcatattgctgcg 1284
            |||||||||||||||||||||||||||||||||||||||||||||||||| ||||| |||
Sbjct: 1872 ccaaagctgtaaacagagcacaaaacagcaaacacttgctccttatggcagattgcagcg 1931


Query: 1285 gcgcaacttgaaaggg-ggagccgggcccgcgtaggaggcaagagccggccgcaaagagc 1343
            ||||||||||||||||  ||||| ||  || ||||||||  ||||||||||||||| | |
Sbjct: 1932 gcgcaacttgaaagggaagagcccgggacgtgtaggaggtgagagccggccgcaaa-atc 1990


Query: 1344 cgctcagcggcattttgtcttcaaaccggccgggtcggatgtggcatttgaagaaagaag 1403
            ||||| || || |||| ||||||||||  | |||||||||||||||||||||||||||||
Sbjct: 1991 cgctcggccgctttttctcttcaaaccctcagggtcggatgtggcatttgaagaaagaag 2050


Query: 1404 gcgtgggtcaataatcaacccgcactttctcctccaaactgctgacgcga--ctcaccgc 1461
            ||||||||||||||||||||||||||||||||||||||||||||||||||  ||||||||
Sbjct: 2051 gcgtgggtcaataatcaacccgcactttctcctccaaactgctgacgcgactctcaccgc 2110


Query: 1462 cttctcagctaagccagctgcagggaaggcc 1492
            |||||||||||||||||||||||||||||||
Sbjct: 2111 cttctcagctaagccagctgcagggaaggcc 2141
```
